# A Dual-Band Bandpass Filter with Wide Upper Stopband Using Stepped-Impedance Resonators and an Integrated Low-Pass Filter

**DOI:** 10.3390/mi17010075

**Published:** 2026-01-06

**Authors:** Liqin Liu, Yuanmo Lin, Qun Chen, Li Zhang, Minhang Weng

**Affiliations:** 1 College of Artificial Intelligence, Putian University, Putian 351100, China; 2 Electronic Information Industry Technology Research Institute of Putian, Putian 351100, China

**Keywords:** wide upper stopband, low loss, stepped impedance resonator (SIR), quality value

## Abstract

In this paper, a dual-band bandpass filter with a wide upper stopband is proposed and designed by integrating stepped-impedance resonators (SIRs) and a low-pass filter. The operating center frequencies of the designed dual-band filter are targeted at 2.5 GHz and 5.35 GHz, respectively, to meet the frequency requirements of typical wireless communication scenarios. Notably, the filter achieves a wide upper stopband ranging from 6.1 GHz to 25 GHz, which can effectively suppress unwanted high-frequency interference signals within this frequency range and avoid mutual interference with other high-frequency communication systems. And it exhibits insertion losses of 0.12 dB (2.5 GHz) and 0.6 dB (5.35 GHz) in its two passbands to ensure minimal useful signal attenuation. The simulation results agree well with the measured results.

## 1. Introduction

The rapid advancement of modern communication systems has spurred significant developments in planar filters. Consequently, the design criteria for wide stopband filters, demanding compact size, high selectivity, and superior stopband performance, have become increasingly stringent.

A key requirement is the effective elimination of spurious harmonics and noise, which necessitates wide stopband filters with excellent out-of-band rejection. Conventionally, wide stopbands are realized by cascading multiple resonators [1,2,3,4]. However, this approach often leads to excessive physical dimensions and significant insertion loss. This has driven many researchers to investigate novel techniques for creating wide stopbands. One popular approach is the Defected Ground Structure (DGS), which achieves a wide stopband by modifying the ground plane. A single DGS unit can typically generate one or two transmission zeros, and advantageous filtering characteristics can be achieved through careful structural optimization [5,6,7]. For instance, Guoan Wu et al. [8] introduced a bandpass filter based on a half-wavelength SIR, employing open stubs and a DGS to shape the stopband and achieve good performance. Nevertheless, DGSs can compromise the ground plane’s integrity and increase the overall size, hindering integration. Deshan Tang et al. [9] developed a dual-passband filter using two Substrate Integrated Defected Ground Structure (SIDGS) resonant units, achieving a wide upper stopband extending to 5.5 f_1_ with low radiation loss. Fuchang Chen [10] designed wide stopband filters and diplexers using Uniform Impedance Resonators (UIRs), generating multiple transmission zeros by tuning the electrical lengths of parallel-coupled lines and stubs to suppress harmonics. Pin Wen et al. [11] designed a dual-band wide stopband filter by analyzing an open-stub-loaded, short-circuited stepped-impedance resonator with hybrid electromagnetic coupling. Minhang Weng et al. [12] created a dual-band filter with stub-loaded stepped-impedance ring resonators, adding rectangular stub-loaded resonators (RSLRs) at the I/O ports for upper stopband suppression. Xueyu Huang et al. [13] fabricated a dual-band filter using a dual-mode dual-composite right/left-handed (D-CRLH) resonator, achieving a stopband up to 4.45 f_1_. Zhang, Y. et al. [14] utilized an inverse design model based on a conditional deep convolutional generative adversarial network (CDC-GAN) to optimize a dual-band filter, achieving a stopband extension to 4.67 f_1_. Mingming Ma et al. [15] proposed a wide stopband dual-band filter based on a self-coupled resonant cavity with asymmetric parallel microstrip lines, extending its stopband to 4.82 f_1_. Weisheng Tang [16] adopted symmetric coupled quarter-wavelength stepped-impedance resonators (SIRs) to design a dual-band ultra-wide stopband filter. By adjusting the feeding position and electrical length ratio of the SIRs, the desired suppression level is achieved without affecting the characteristics of the fundamental passband, exhibiting excellent performance. Yijun Li et al. [17] utilized a slot-loaded substrate-integrated waveguide (SIW) cavity design with microstrip line feeding. Two quarter-wavelength resonators were introduced into the upper and lower transverse slots, which were, respectively, coupled with two shorted patches, achieving a −20 dB stopband range of up to 2.86 f_1_. In Bowei Liu et al.’s study [18], the top layer of the filter employed hook-shaped microstrip feeding lines (HSMFLs) composed of stepped-impedance variation microstrip lines (SIVMLs) and folded microstrip lines with an enhanced coupling effect at the ends. A T-junction resonator was arranged on the second layer, thus realizing a bandpass filter based on low-temperature co-fired ceramic (LTCC) technology with an upper stopband width of 3.35 f_1_, which exhibited excellent performance. Despite these advances, the stopbands in many existing designs are insufficient to suppress all spurious harmonics, and achieving harmonic suppression across an ultra-wide frequency range remains a formidable challenge.

In this work, a dual-band filter featuring a wide upper stopband is proposed. The filter is composed of a stepped-impedance resonator (SIR) section combined with a low-pass filter (LPF). The dual-band response is achieved through interdigital coupling between the SIRs. Subsequently, the integrated LPF suppresses the upper passband of the dual-band filter, resulting in a wide upper stopband capable of effectively rejecting high-frequency interference.

## 2. Design Process

**Figure 1:**
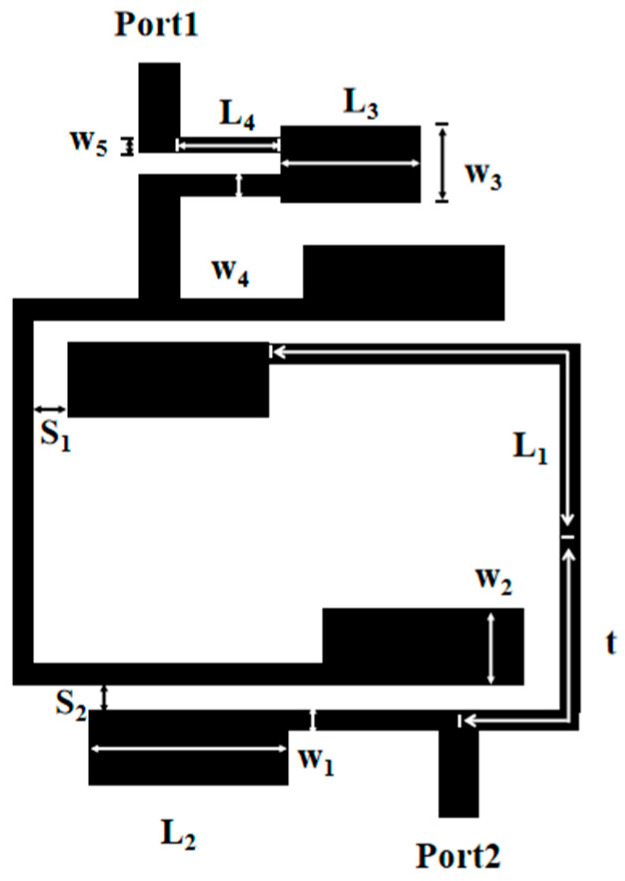
Geometrical diagram.

Figure 1 presents the geometric configuration of a dual-band filter with upper wide stopband. This BPF is capable of meeting WLAN standards while concurrently delivering a wide upper stopband, high band selectivity, and low insertion loss.

The substrate employed here is Rogers RT/duroid 5880, characterized by a thickness of 0.787 mm, a relative dielectric constant (ε_r_) of 2.2, and a loss tangent of 0.0009. The dual-band BPF prototype primarily consists of two key components: a set of cross-coupled stepped-impedance resonators (SIRs) and a low-passband filter. Input and output (I/O) lines are coupled to the cross-coupled SIRs via taps on both sides. As a result, two signal transmission paths are formed between the input and output ports—a design feature is the intentional introduction of the cross-coupling effect, which is critical to the performance of this dual-band bandpass filter. The simulation is conducted using Ansys HFSS 2023 R1, a high-frequency structural simulation tool suitable for microwave passive components. Regarding boundary conditions, the upper surface of the substrate (microstrip structure) adopts a Radiation Boundary to avoid boundary reflection; the lower surface (ground copper foil) uses a Perfect E Boundary to simulate a complete ground plane; and the substrate sides employ a Perfect H Boundary to mimic infinite extension. For port configuration, the input and output ports are Wave Ports, with parameters matching the microstrip lines, a characteristic impedance of 50 Ω, and excitation in TEM mode.

### 2.1. Dual-Band Filter Design

The dual-band filter proposed in this work is targeted for operation at 2.5 GHz and 5.35 GHz. For its two passbands, the 3 dB fractional bandwidths (FBWs) are configured as follows: the first passband (centered at 2.5 GHz) has an FBW1 of 34.8%, while the second passband (centered at 5.35 GHz) features an FBW2 of 12.1%. Notably, both passbands share an identical passband ripple of 0.01 dB.

**Figure 2:**
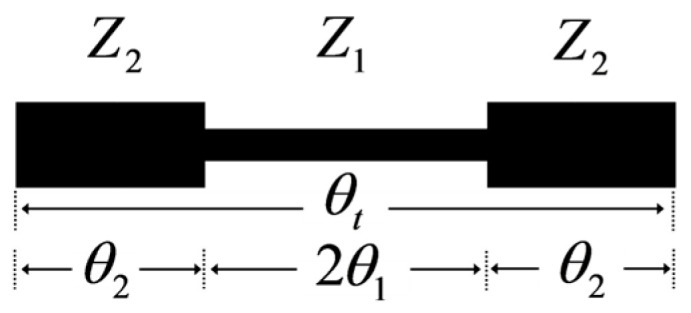
Schematic of the stepped-impedance resonator.

Prior to analyzing the filter’s overall performance, it is necessary to first discuss the resonant behavior of the stepped-impedance resonator (SIR) illustrated in Figure 2. This SIR is structured by cascading two high-impedance segments (denoted as *Z*_2_) with a single low-impedance segment (denoted as *Z*_1_). The impedance ratio (*R*)—a key parameter of the SIR—is defined as *R* = *Z*_2_/*Z*_1_. The input admittance (Y*_in_*) of the SIR is expressed by the equation below:

(1) $$Y_{in}=\frac{2(R\tan\theta_{1}+\tan\theta_{2})\cdot (R-\tan\theta_{1}\cdot \tan\theta_{2})}{R(1-\tan^{2}\theta_{1})\cdot (1-\tan^{2}\theta_{2})-2(1+R^{2})\cdot \tan\theta_{1}\cdot \tan\theta_{2}}$$

The resonant conditions of the SIR can be derived when its Y*_in_* equals zero. Additionally, by adjusting *R* and *α* of the SIR, the positions of its higher-order resonant modes can be tuned—either bringing these modes closer to each other or spacing them farther apart. The definition of the length ratio (*α*) is given as follows:

(2) $$\alpha =\frac{\theta_{2}}{\theta_{1}+\theta_{2}}=\frac{2\theta_{2}}{\theta_{t}}$$

**Figure 3:**
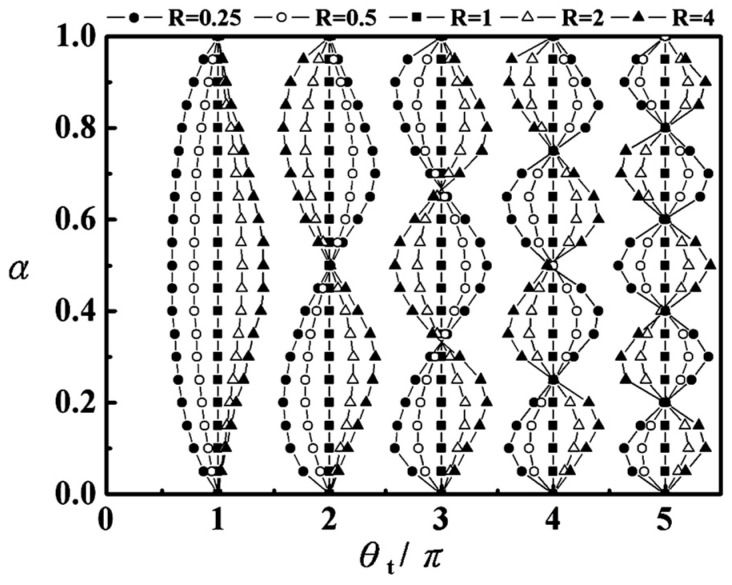
Fundamental and higher-order resonant modes as function of the length ratio *α* and impedance ratio *R* = 0.25, 0. 5, 1, 2, and 4.

The introduction of *α* enriches the resonant mode characteristics of the SIR, thereby providing greater design flexibility for the filter. Here, *θ_t_* represents the total electrical length of the SIR. When Equation (2) is substituted into Equation (1), multiple resonant modes—whose properties are dependent on both the *R* and *α*—can be derived, and these modes are visualized in Figure 3.

Notably, in the non-stepped-impedance scenario (i.e., when *R* = 1, which is equivalent to *α* = 0 or *α* = 1), the relationship *θ_t_* = *n*π holds. This result indicates that the nth resonant mode of the resonator is excited when its total length corresponds to *n* times a half-wavelength.

It can be clearly observed that the spacing between certain higher-order resonant modes and the fundamental resonant mode (i.e., whether they are far apart or close to each other) depends on the selection of the *R* and *α*. Therefore, the same fundamental SIR structure can be extended to design dual-band, three-band, or even four-band filters, highlighting the scalability and flexibility of this approach for more complex multi-band applications. Thus, by appropriately choosing *R* and *α* of the SIR, it is feasible to achieve a dual-band response. Following the above guidelines, we mapped the requirements for the two target center frequencies onto Figure 3. By adjusting the impedance ratio *R* and length ratio *α*, the positions of high-order resonant modes can be flexibly tuned (Figure 3). For example, when *R* = 0.5 and *α* = 0.26, the resonant modes are exactly at 2.5 GHz (fundamental mode) and 5.35 GHz (second-order mode). In contrast, the resonant modes of uniform impedance resonators (UIRs) are fixed at *n* × fundamental frequency (e.g., 2.5/5/7.5 GHz), which cannot match the 5.35 GHz requirement. This specific value allows the filter to attain two key performance traits at the same time: a dual-band operating response suitable for WLAN uses, and an extensive stopband. The electrical sizes of the SIR at the operating resonance frequencies that are calculated using the free-space wavelength are 120 mm at 2.5 GHz and 56 mm at 5.35 GHz. A single SIR can provide two or more resonant modes eliminating the need for multiple independent resonators. The total length of the SIR in this work is 39.5 mm, which is shorter than the length of two independent UIRs. Also, there are some alternative routes to compact multiband resonators [19], for example, using dielectric materials with very high relative permittivity as the core resonator and relying on subwavelength resonators (e.g., split-rings, split-loops) to confine electromagnetic energy at the subwavelength scale. Constructed into the designed filter are high-impedance line sections (*Z*_1_ = 100 Ω) featuring a 0.7 mm strip width, as well as low-impedance line sections (*Z*_1_ = 50 Ω) with a 2.4 mm strip width. In the initial design stage, the target performance specifications were first defined. Based on these specifications, the lumped-element values of the low-pass prototype filter were calculated, yielding *g*_1_ = 0.90467 and *g*_2_ = 1.25868 (where *g_n_* denotes the *n*-th lumped-element value for *n* = 1,2) [20]. Once these lumped-element values were obtained, the required coupling matrices M and external quality factor (*Q_e_*) of the bandpass filter (BPF) were derived. This derivation was conducted on the basis of coupling theory, while also adhering to the standard design protocol presented in reference [20].

(3) $$M_{ij}^{I}=\left[\begin{array}{cc} 0 & 0.326\\ 0.326 & 0 \end{array}\right]\;\mathrm{at}\;2.5\;\mathrm{GHz}$$

and

(4) $$M_{ij}^{II}=\left[\begin{array}{cc} 0 & 0.113\\ 0.113 & 0 \end{array}\right]\;\mathrm{at}\;5.35\;\mathrm{GHz}$$

$$Q_{e}^{I}=3.27\;\mathrm{and}\;Q_{e}^{II}=9.41$$

where *M_ij_* denotes the coupling coefficient, with $M_{12}=M_{21}=FBW/\sqrt{g_{1}g_{2}}$ and $Q_{e}=g_{1}g_{2}/FBW$.

To realize the pre-set band performance, the coupling levels between adjacent SIRs should be suitably selected to correspond with the intended coupling matrix *M*. Particularly, the coupling level between Resonator 1 and Resonator 2 is primarily shaped by the spacing S_2_; hence, S_2_ is adjusted to regulate the coupling coefficient *M*_12_. In addition, the external quality factor *Q_e_* of the dual-band BPF is affected by the distance t—that is to say, varying the distance t makes it possible to adjust *Q_e_*. It should be emphasized that the computed coupling between resonators can be distinguished by two main resonant frequencies. These frequencies arise from the splitting of the original resonance condition, which is induced by electromagnetic coupling. Based on this principle, the simulated coupling coefficient M is computed as follows [20]:

(5) $$M=\frac{f_{H}^{2}-f_{L}^{2}}{f_{H}^{2}+f_{L}^{2}}$$

**Figure 4:**
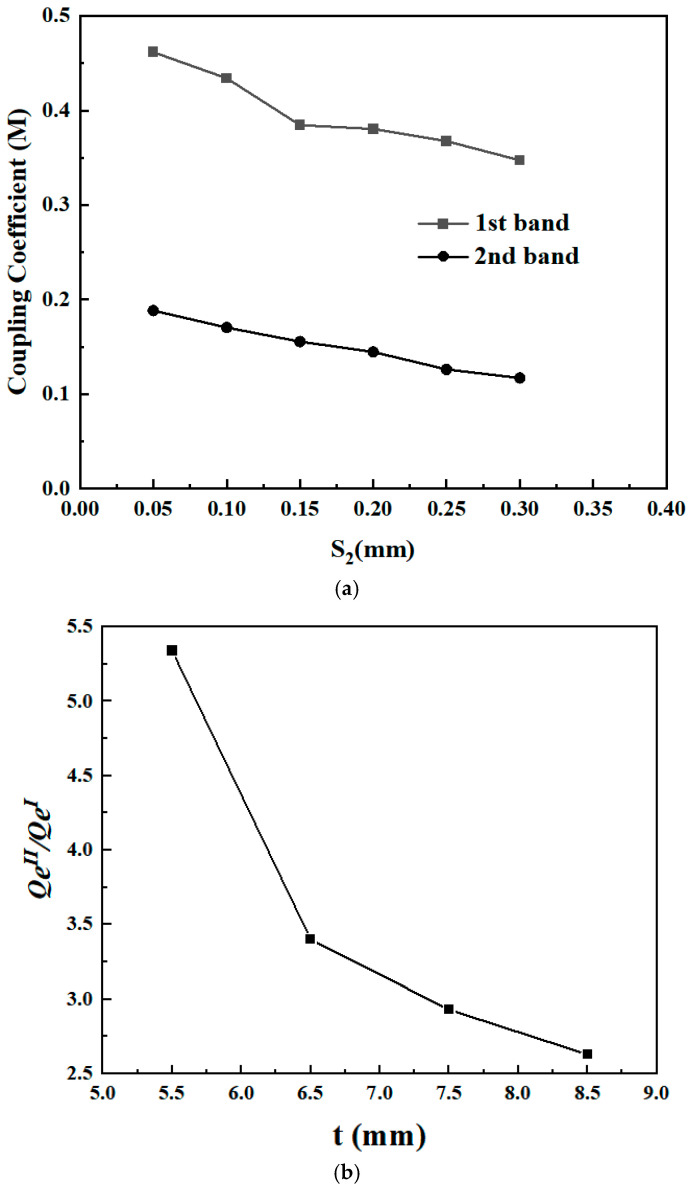
(**a**) Coupling coefficient *M* and (**b**) external quality *Q_e_* of the dual-band BPF as shown in Figure 1 for 1st passband and 2nd passband simultaneously.

The calculated quality factor values between the resonators can be characterized by the two threshold resonant frequencies. Accordingly, the simulated external quality factor *Q_e_* is computed as follows [20]:

(6) $$Q_{e}=\frac{f_{0}}{f_{H\;3dB}-f_{L\;3dB}}$$

where *f_H_*_3_*_dB_* denotes the higher frequency of the 3 dB fractional bandwidth (FBW), and *f_L_*_3_*_dB_* denotes the lower one. The coupling coefficient *M* and external quality factor *Q_e_* of the dual-band BPF, as illustrated in Figure 4, were calculated using a full-wave electromagnetic (EM) simulator.

**Figure 5:**
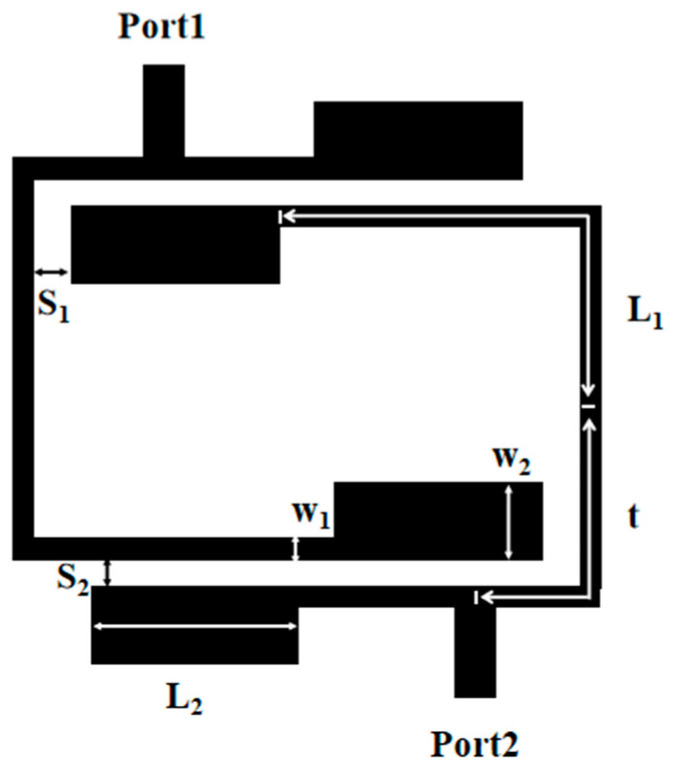
Structure diagram of the dual-frequency filter.

**Figure 6:**
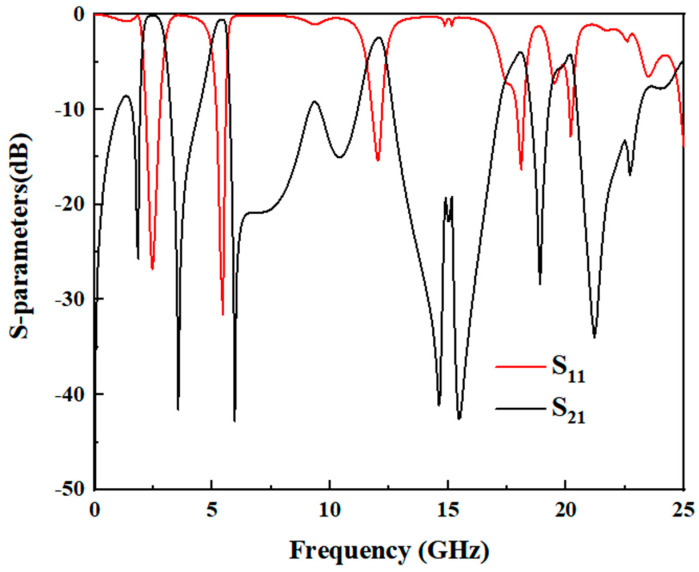
Simulation S-parameter diagram of the dual-frequency filter.

Figure 5 is the structure diagram of the dual-frequency filter. Optimal simulation of the filter’s performance was carried out via a full-wave electromagnetic (EM) simulator, with subtle structural tweaks applied over the course of the process. The simulation results are presented in Figure 6, using the optimized structural parameters: L_1_ = 14.65 mm, w_1_ = 0.7 mm, L_2_ = 5.1 mm, w_2_ = 2.4 mm, t = 7.5 mm; S_1_ = 0.3 mm, and S_2_ = 0.25 mm. The dual-band filter delivers dual-band filtering performance with center frequencies at 2.5 GHz and 3.53 GHz, boasting 3 dB bandwidths of 34.8% and 12.1% for its respective passbands. Employing a 0-degree feed arrangement for both input and output, the filter creates transmission zeros at 1.83 GHz, 3.56 GHz, and 6 GHz, thus achieving high selectivity. Nevertheless, it has been noted that a significant number of harmonics from higher-order modes manifest within the 6.1 GHz to 25 GHz range.

### 2.2. Wide Upper Stopband Design

**Figure 7:**
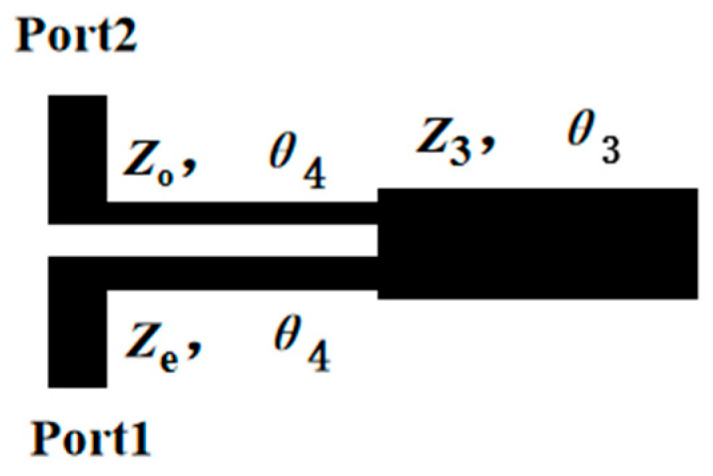
The low-pass filter used in this design.

**Figure 8:**
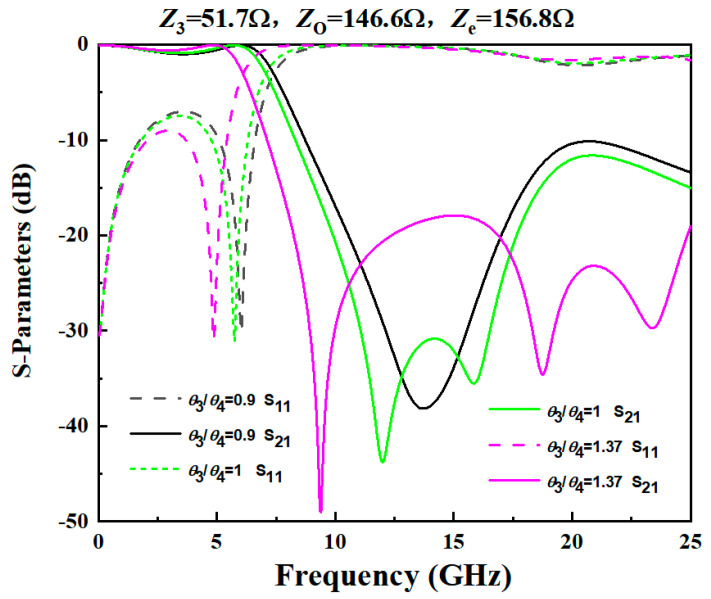
Comparison chart of S-parameters of low-pass filters under different electron length ratios.

A wide upper stopband is essential in multi-service wireless systems (e.g., WLAN, 5G, satellite communications) to prevent interference from higher-frequency signals and harmonics generated by the filter itself or other active components in the RF front-end. To implement the wide upper stopband of the proposed dual-band filter, it is necessary to suppress harmonics at 6.1 GHz and 25 GHz. Additionally, the rejection of unwanted interference in the higher frequency range (6.1 GHz to 25 GHz), as indicated in Figure 6, needs to be improved. This is achieved by incorporating a low-pass filter (LPF), as depicted in Figure 7. The LPF is designed to provide an adjustable stopband for effective high-frequency signal suppression. The structure of the LPF consists of a pair of coupled lines and a shunt open-circuited resonator, realized as a high-impedance–low-impedance–high-impedance transmission line configuration. As demonstrated in [21], a wide stopband with effective out-of-band rejection can be obtained when the lengths of the coupled lines and the shunt resonator are approximately a quarter-wavelength (λg/4) at a specific center frequency. Figure 8 presents a comparative analysis of the LPF’s S-parameters for different electrical length ratios (*θ*_2_/*θ*_1_). The analysis reveals that the sharpest attenuation slope is achieved when *θ*_3_/*θ*_4_ = 1.37, which offers superior suppression of adjacent channels and spurious signals.

### 2.3. Design of the Dual-Band Bandpass Filter with a Wide Upper Stopband

**Figure 9:**
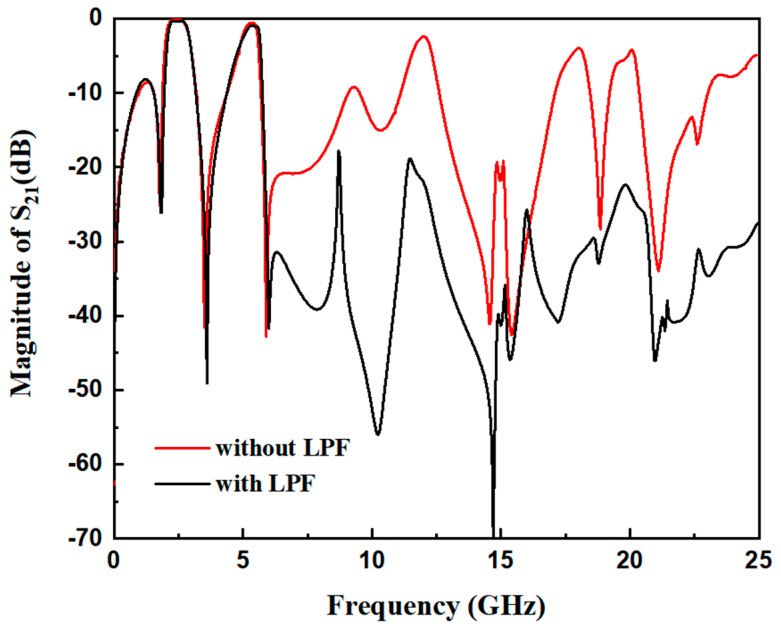
Comparison chart of S_21_ dual-frequency filter with low-pass filter and dual-frequency filter without low-pass filter.

To achieve the desired wide stopband, the low-pass filter (LPF) is integrated with the input transmission line of the dual-band filter, as depicted in the final layout in Figure 1. The final physical dimensions have been optimized as follows: L_1_ = 14.65 mm, w_1_ = 0.7 mm, L_2_ = 5.1 mm, w_2_ = 2.4 mm, L_3_ = 3.36 mm, w_3_ = 2.3 mm, L_4_ = 2.45 mm, w_4_ = 0.2 mm, w_5_ = 0.25 mm, t = 7.5 mm, S_1_ = 0.3 mm, and S_2_ = 0.25 mm. As shown in Figure 9, the integration of the LPF significantly enhances the filter’s performance by providing a lower insertion loss and a substantially wider upper stopband. The filter exhibits an insertion loss of 0.12 dB in the first passband and 0.6 dB in the second passband. Notably, the upper stopband is extended to cover the frequency range from 6.1 GHz to 25 GHz.

## 3. Experimental Test Results

**Figure 10:**
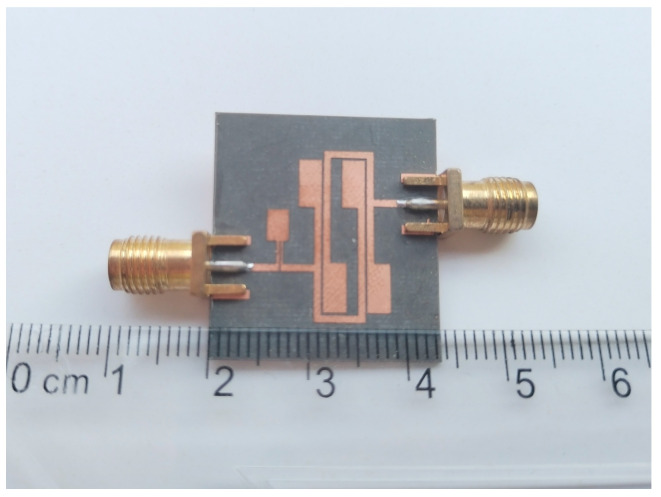
Photograph of the fabricated dual-band BPF with wide upper stopband.

**Figure 11:**
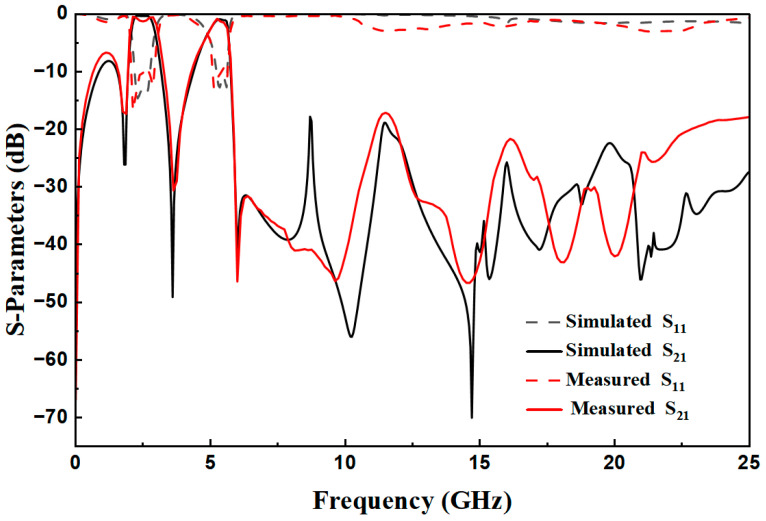
Comparison between simulation and actual measurement.

The designed filter is fabricated and then measured by a Network Analyzer. A photograph of the fabricated dual-band BPF with a wide upper stopband is shown in Figure 10. The dimensional parameters of the filter are as follows: L_1_ = 14.65 mm, w_1_ = 0.7 mm, L_2_ = 5.1 mm, w_2_ = 2.4 mm, L_3_ = 3.36 mm, w_3_ = 2.3 mm, L_4_ = 2.45 mm, w_4_ = 0.2 mm, w_5_ = 0.25 mm, t = 7.5 mm, S_1_ = 0.3 mm, and S_2_ = 0.25 mm. The input and output ports are connected via a 50 Ω broadband transmission line with a width of 0.8 mm. Additionally, the whole size is only 15.46 mm × 16.46 mm, i.e., approximately ] 0.16 λg × 0.17 λg, where λg is the guided wavelength at the first frequency. The simulated results and the measured results are shown in Figure 11. The measured results have a low insertion loss (|S_21_|) less than 0.12 dB, a bandwidth of 34.8%, and a *Q_e_* value of 3.27 for 2.5 GHz, and a low insertion loss (|S_21_|) less than 0.6 dB, a bandwidth of 12.1%, and a *Q_e_* value of 9.41 for 5.35 GHz. And the range of the upper stopband is from 6.1 GHz to 25 GHz. The transmission zeros can be obviously introduced on the two-side skirt of each passband, which is due to the coupling effect between two SIRs, causing the multi-path propagation mode and 0-degree feed. The transmission zeros significantly improve the selectivity of the fabricated dual-band BPF with a wide upper stopband. The mean absolute error between simulated and measured curves in Figure 11 can be calculated as 0.57 dB. The minor discrepancy primarily stems from substrate etching tolerance (±0.05 mm) and SMA connector insertion loss (~0.1 dB), confirming good consistency between simulation and measurement.

Table 1 shows the comparison of major performance characteristics of the proposed filter against data presented in other research reports. The proposed filter achieves lower insertion loss (0.12 dB at 2.5 GHz, 0.6 dB at 5.35 GHz), outperforming prior studies [11,15], and a wider upper stopband (6.1–25 GHz, 10 f_1_), surpassing [13,15] to suppress harmonics below 10f_1_. Structurally, it directly integrates “cross-coupled SIRs” with a “λg/4-loaded LPF” (not cascaded), reducing size to 63% of [12]. Applicable to WLAN dual bands, it suppresses 5G millimeter-wave (24/28 GHz) and satellite communication (12–18 GHz) interference, enabling direct use in RF front-ends to simplify systems.

## 4. Conclusions

This paper designs a dual-band filter with a wide upper stopband and implements it by using stepped-impedance resonators and a low passband filter. Analysis of the odd and even modes reveals the resonant frequency distribution of the stepped-impedance resonators. The proposed filter’s design entails two key steps: first, developing the dual-band filter, and then integrating it with a low passband filter to achieve harmonic suppression. The resulting filter features dual passbands centered at 2.5 GHz and 5.35 GHz, with 3 dB bandwidths of 34.8% and 12.1%, respectively. It has three transmission zeros, ensuring excellent selectivity. Notably, it achieves |S_21_| attenuation exceeding 20 dB across 6.1 GHz to 25 GHz, demonstrating effective harmonic suppression up to ten times the fundamental frequency. And the designed filter is also compact. This proposed dual-band BPF with a wide stopband is well-suited for WLAN systems.

## Figures and Tables

**Table 1:** Comparison of major performance characteristics of the proposed filter against data presented in other research reports.

	1st/2nd Passbands (GHz)	|S_21_| (dB)	FBW (%)	Upper Stopband	Circuit Size (mm^2^) (λg^2^)
11	1.51/3.68	1.55/1.63	4.25/2.6	6.4 f_1_	0.0065
12	2.4/4	1.4/1	8/39	8.3 f_1_	0.0432
13	3.5/5.8	0.73/1.43	18/8.3	4.45	0.010282
14	3/5	1.19/1.77	17.9/4.1	4.67	0.26
15	2.34/4.72	1.05/1.86	15.8/8.33	4.82	0.0224
Proposed Filter	2.5/5.35	0.12/0.6	34.8/12.1	10 f_1_	0.0272

## Data Availability

All the material conducted in the study is mentioned in article.
